# Sensitivity of PCR in conjunctival swab samples for the diagnosis of canine visceral leishmaniasis: a systematic review and meta-analysis

**DOI:** 10.1590/S1984-29612023063

**Published:** 2023-11-27

**Authors:** Mariana Fagundes Bento, Gabriela Scarpin de Souza, Bruno Serpa Vieira, Ângela Adamski da Silva, Felipe da Silva Krawczak, Veridiana Maria Brianezi Dignani de Moura

**Affiliations:** 1 Setor de Patologia Animal, Departamento de Medicina Veterinária, Escola de Veterinária e Zootecnia, Universidade Federal de Goiás – UFG, Goiânia, GO, Brasil; 2 Departamento de Zootecnia, Instituto Federal de Mato Grosso – IFMT, Alta Floresta, MT, Brasil; 3 Laboratório de Patologia Molecular, Instituto de Ciências Biológicas, Universidade Federal de Goiás – UFG, Goiânia, GO, Brasil; 4 Setor de Medicina Veterinária Preventiva, Departamento de Medicina Veterinária, Escola de Veterinária e Zootecnia, Universidade Federal de Goiás – UFG, Goiânia, GO, Brasil

**Keywords:** Evidence-based medicine, Leishmania infantum, PCR, sensitivity, zoonotic disease, Medicina baseada em evidências, Leishmania infantum, PCR, sensibilidade, doença zoonótica

## Abstract

To compare the sensitivity of conjunctival swab (CS) and conventional samples (blood, spleen, liver, lymphoid and cutaneous tissue) in the diagnosis of canine visceral leishmaniasis (CVL) by polymerase chain reaction (PCR), a systematic review and meta-analysis was carried out using PubMed, Science Direct, Scopus, Web of Science, VHL/BVS (Virtual Health Library), CAPES, and Scielo databases. Articles published from 2002 to 2022 were considered and the review was updated in Jul 2023. From the total of 371 identified studies, 8 met all the eligibility criteria and were included in this review. Data from 658 CVL-positive dogs and 2541 PCR results were considered. Using a random effect model, data on the sensitivity of the test was compared between intervention (CS samples) and comparison (all the other samples) groups. Overall, the use of CS in the PCR diagnosis of CVL produced 12% higher sensitivity (p=0.013) in the test than all the other samples in combination. The animals' clinical condition did not influence (p>0.142) this overall result. However, when CS was individually compared to each of the conventional samples, the consistent result was observed (p=0.012) only in the CS versus bone marrow comparison. Given their rapid acquisition, minimal invasiveness, and lower cost relative to conventional samples, CS samples present a promising alternative for the molecular diagnosis of CVL.

## Resumo

Para comparar a sensibilidade do *swab* conjuntival (SC) e amostras convencionais (sangue, baço, fígado, tecido linfoide e cutâneo) no diagnóstico da leishmaniose visceral canina (LVC), pela reação em cadeia da polimerase (PCR), uma revisão sistemática e metanálise foi realizada nas bases de dados PubMed, Science Direct, Scopus, Web of Science, BVS (Biblioteca virtual da saúde), CAPES e Scielo. Artigos publicados entre 2002 e 2022 e a revisão foi atualizada em julho de 2023. Do total de 371 estudos identificados, 8 preencheram todos os critérios de elegibilidade e foram incluídos nesta revisão. Dados de 658 cães LVC-positivos e 2.541 resultados de PCR foram considerados. Usando-se um modelo de efeito aleatório, os dados sobre a sensibilidade do teste foram comparados entre os grupos de intervenção (amostras de SC) e de comparação (todas as outras amostras). No geral, o uso de SC no diagnóstico por PCR de LVC produziu sensibilidade 12% maior (p=0,013) no teste do que todas as outras amostras combinadas. A condição clínica dos animais não influenciou (p>0,142) neste resultado global. Porém, quando o SC foi comparado individualmente com cada uma das amostras convencionais, o resultado consistente foi observado (p=0,012) apenas na comparação do SC *versus* medula óssea. Dada a sua rápida aquisição, invasividade mínima e menor custo em relação às amostras convencionais, as amostras de SC apresentam uma alternativa promissora para o diagnóstico molecular da LVC.

## Introduction

Canine visceral leishmaniasis is a zoonotic disease caused by the protozoan parasite *Leishmania infantum*. Accurate and timely diagnosis of canine visceral leishmaniasis is crucial for implementing effective control measures to prevent the spread of the disease (Silva et al., 2021). The current gold standard tests used for the diagnosis of canine visceral leishmaniasis involve a combination of methods for direct detection of the parasite (DPP), such as microscopy of bone marrow aspirate or lesioned tissue, and confirmatory serological tests based on techniques such as immunochromatographic test, indirect immunofluorescence (IFI) and enzyme-linked immunosorbent assay (ELISA) (Duthie et al., 2018).

The diagnosis of canine visceral leishmaniasis (CVL) is sometimes complex, since several factors directly affect the accuracy of the tests considered gold standard for confirmation of the disease. However, variables involved in the tests, such as the antigen type used, the clinical condition of the dog, and the possibility of cross-reactions, may lead to false-negative and false-positive results (Silva et al., 2021; Rocha et al., 2019). In-house tests using DPP and ELISA demonstrated cross-reactivity rates of 44% and 22%, respectively, with *Babesia canis* (Laurenti et al., 2014).

A total of 975 dogs from an endemic region were evaluated using the rapid immunochromatographic test (TR-DPP), ELISA, and real-time polymerase chain reaction (qPCR) for the diagnosis of canine visceral leishmaniasis (CVL). In a study by Lopes et al. (2017), DPP-negative dogs were tested by qPCR using blood and lymph node aspirates, 174/887 (19.6%) tested positive in at least one sample. In a subsequent sampling of 115 cases, DPP-negative dogs were tested by qPCR using blood, lymph node, and conjunctival swab (CS) samples, and 36/79 (45.6%) tested positive in at least one sample.

The use of molecular techniques minimizes false negative results, and the polymerase chain reaction (PCR) is the most widely used test (Duthie et al., 2018). This method enables the identification and amplification of parasite’s DNA sequences, allowing for the analysis of different types of samples, including those from lymph nodes, bone marrow, blood, spleen and liver (Lopes et al., 2016; Thakur et al., 2020).

The accuracy of PCR from those conventional samples in the diagnosis of CVL is well accepted both in the literature and clinical practice. However, the invasiveness of the procedures used to obtain them is frequently questioned, especially by dog owners during screening tests. Therefore, samples obtained from urine, conjunctival, nasal, and oral swabs have been evaluated as alternatives to the conventional ones, as they are minimally invasive and do not require the collection of tissue fragments (Ferreira et al., 2012; Lombardo et al., 2012).

Ocular manifestations may occur in dogs infected with *Leishmania infantum*, including blepharitis, conjunctivitis, uveitis and hyphema (Di Pietro et al., 2016). The structures attached to the eye may also be affected and positive animals may have eyelid nodules, periocular alopecia and keratoconjunctivitis sicca (Ferreira et al., 2012; Di Pietro et al., 2016). PCR-based diagnostic methods using CS samples have been published (Ferreira et al., 2012; Duthie et al., 2018), but the sensitivity and specificity of the test for the diagnosis of CVL, in both symptomatic and asymptomatic dogs, are still uncertain. Therefore, using evidence-based medical approaches, this study focused on a systematic review of the literature and meta-analysis to determine whether the use of CS generates different sensitivity than conventional samples (blood, spleen, liver, lymphoid and cutaneous tissue) in the diagnosis of CVL by PCR in positive animals.

## Material and Methods

### Research question and study selection

The research question to be answered was: In PCR-based diagnostic methods for CVL, is the sensitivity of the test using CS samples lower than that obtained when using conventional samples, including blood, spleen, liver, lymphoid and cutaneous tissue? To answer this question, a systematic review of the literature was carried out between 2002 and 2022, and was updated in Jul 2023. Applying principles from the PICO (population: dogs with canine visceral leishmaniasis; intervention: CS using PCR; comparison: PCR using samples of blood tissue, lymphoid tissue (bone marrow and lymph node), or cutaneous tissue; outcomes: total number of tested and positive samples) strategy, the following search algorithm was constructed, using both MeSH descriptors and keywords frequently adopted in the area: *(dogs OR canines) AND (leishmaniasis OR leishmania OR infantum) AND (“polymerase chain reaction” OR PCR) AND conjunctiva*. The search algorithm was then applied in MEDLINE (PubMed), ELSEVIER (Science Direct and Scopus), WoS (Web of Science), VHL/BVS (Virtual Health Library), and Scielo (Scientific Electronic Library Online) scientific databases, using no automatic filters. The Theses Database of Brazil’s Federal Agency for the Support and Improvement of Higher Education (CAPES) was also consulted. Results were exported to a reference manager software (Zotero 6.0.23) where an initial screening process was conducted.

Duplicated studies were automatically excluded and then titles and abstracts were read and screened. Sequentially the selected studies underwent through a full-text reading, and those that met the following inclusion criteria, were included in the meta-analysis: i) primary studies, published in the last ten years, in English, Spanish or Portuguese; ii) studies involving dogs of any age, breed and sex, with visceral leishmaniasis confirmed by a reference test); iii) studies that compared the sensitivity of conventional PCR results using CS with that of conventional samples (e.g. blood, spleen, liver, lymphoid and cutaneous tissue). Studies that did not meet all the aforementioned criteria were not included in this review.

After that, bibliographic references cited in the included articles were analyzed in order to ensure complete scan of the available scientific literature on the proposed topic. The results of the systematic review and meta-analysis were presented following the recommendations of Preferred Reporting Items for Systematic Reviews and Meta-Analyses (PRISMA) guidelines (Page et al., 2021).

### Data extraction and analysis

A standardized clinical form with the pre-defined eligibility criteria and other information of interest was used as a guide for data extraction. The extracted data included, for each study, complete reference and country of development, number and clinical condition of the assessed dogs, reference test used for CVL confirmation, PCR results for all the assessed samples, including target genes and type/origin of samples.

Regarding the quantitative variable of interest, dichotomous data were extracted, characterized by the total number of samples analyzed by PCR and the total number of positive samples for each sample type/origin. The PCR sensitivity (number of positive results/number of analyzed samples) was then calculated, in each study, for each sample type/origin and animal’s clinical condition. The non-conjunctival samples were then combined into a single group (comparison group), in order to compare their pooled PCR sensitivity with the pooled PCR sensitivity in the CS samples (intervention group). Finally, the risk of bias in each included study was assessed using the Quality Assessment of Diagnostic Accuracy Studies tool (QUADAS-2) (Reitsma et al., 2009).

For meta-analysis, risk difference (RD) was adopted as the measure of the effect size, allowing the objective comparison of PCR sensitivities between comparison and intervention groups. The RD calculation followed the formula: *RD = PCR sensitivity in CS (intervention group) – PCR sensitivity in non-conjunctival samples (comparison group)*. For each RD, 95% confidence intervals were calculated and individual weights were assigned to each study based on the inverse variance method, meaning that studies with lower variance (greater number of samples) received higher weight.

A weighted average of RDs was calculated and their significance was tested by the Z test, using a random effects model. Heterogeneity between studies was quantified by the I^2^ index and its significance was tested by the Chi^2^ test. In case of significant heterogeneity, subgroup analysis was performed in order to verify the influence of animals’ clinical condition and sample type/origin on the overall result. Studies that did not provide data on the presence or absence of clinical signs in the assessed population were grouped as “not reported”. A significance level of 5% (p≤0.05) was adopted in all steps.

### Meta-analysis robustness and results presentation

Robustness of meta-analysis results were graphically assessed by a sensitivity analysis, plotting each RD against its correspondent standard error (funnel plot). A homogeneous distribution of RDs on both sides of the funnel plot was considered the absence of publication bias. Also, funnel plot asymmetry was formally tested using Egger`s regression (Egger et al., 1997).

Studies whose risk difference fell outside the normality pyramid of the funnel plot were considered potential outliers, and their influence on the overall result was tested by excluding them individually from the analysis. Any significant change in the overall result led to the removal of the study from the analysis; otherwise, the study was maintained. Review Manager 5.4.1 and Jamovi 2.3.19.0 software were used for all the statistical analysis and graphic synthesis of results.

## Results

### Systematic review and data set characterization

As for the databases from which the studies were retrieved, 106 (28.6%) came from PUBMED, 105 (28.3%) from Science Direct and 89 (24.0%) from CAPES. In addition, 30 (8.1%), 17 (4.6%), and 13 (3.5%) papers were retrieved from SCOPUS, BVS and Scielo, respectively. The fewest results (n=11, 2.9%) were obtained from Web of Science.

**Figure 1:**
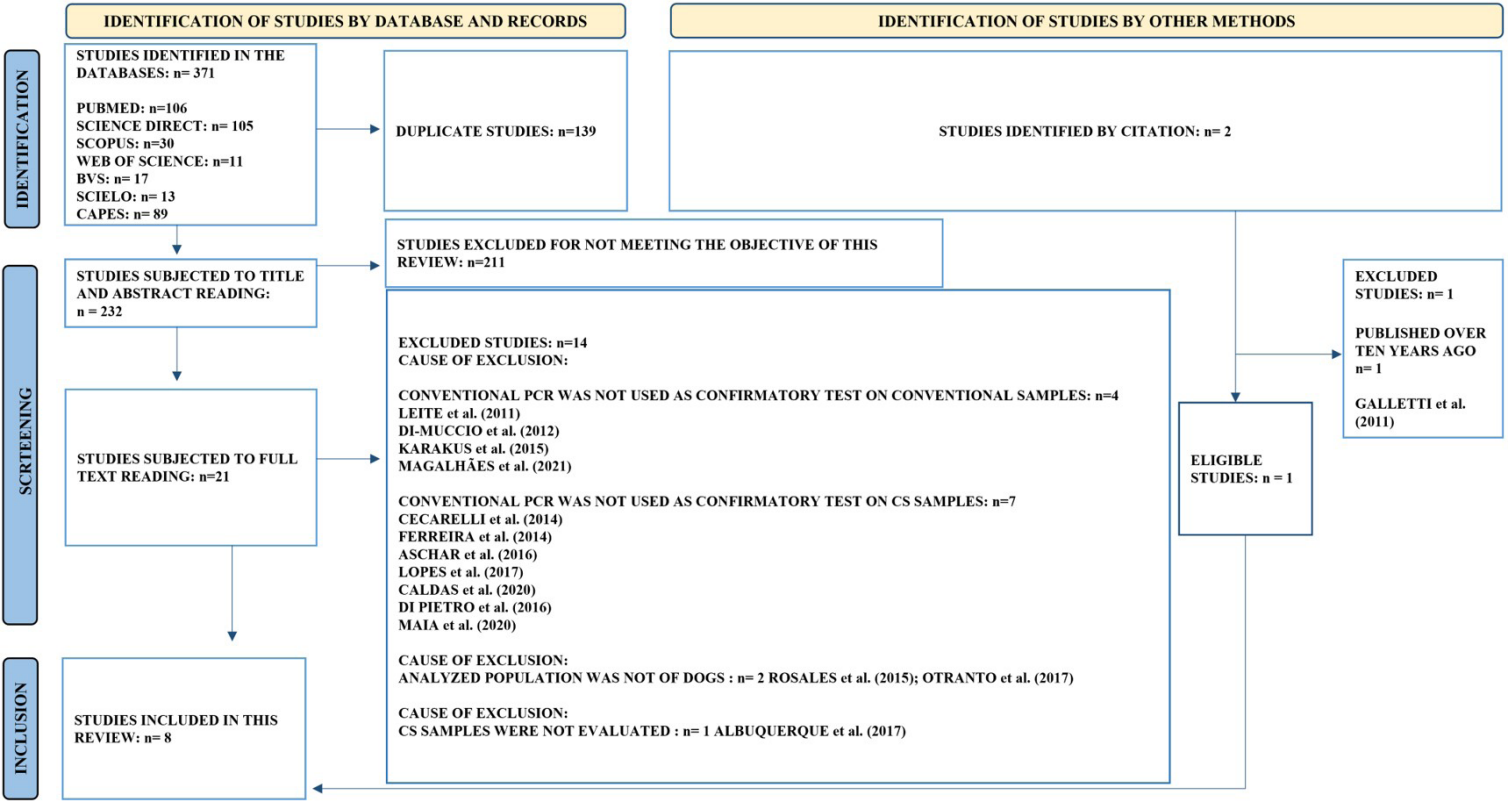
Adapted PRISMA 2020 flowchart showing the process of identification, screening and selection of evidence in the PCR molecular diagnosis of canine visceral leishmaniasis using samples obtained by conjunctival swab from dogs. Adapted from Page et al. (2021). CS= Conjunctival swab; CVL= canine visceral leishmaniasis.

The search for primary studies in scientific databases resulted in 371 studies. After excluding duplicates (n=139) and those screened based on the reading of their titles and abstracts (n=211), 21 studies proceeded to full-text read. During this last screening step, main reasons for exclusion were: conventional PCR was not used as confirmatory test on conventional samples (n=4), conventional PCR was not used as confirmatory test on CS samples (n=7), analyzed population was not of dogs (n=2), CS samples were not evaluated (n= 1). After which, only seven studies met all the eligibility criteria and were included in this meta-analysis. In addition, an analysis of bibliographic references on these seven included studies led to the retrieval of two studies of interest, but only one of them was considered eligible for the meta-analysis. Therefore, our whole dataset comprised eight primary studies. A flowchart showing all the quantitative results of the systematic review processes is presented in Figure 1, where the main causes of exclusion of all studies that were submitted to the full-text reading can also be consulted.

Of the eight studies that met all the eligibility criteria, five were developed in Brazil, while three were carried out in Europe – two in Germany and one in Italy. Beyond the CS used in all the studies (n=8), other types of samples were assessed by authors such as bone marrow (n=5), blood (n=5), lymphoid tissue (n=3) and cutaneous tissue (n=3). To provide a more comprehensive understanding of the set of included studies, an individual approach (by study) of all qualitative characteristics generically described in this section is presented in Table 1.

**Table 1:** References and characteristics of the studies selected for this systematic review.

**Author/year of publication**	**Location of study**	**Sensitivity of the samples evaluated by tissue (Positive Samples/Total Evaluated)**	**Molecular target of PCR in conventional samples**	**Molecular target of PCR in CS**	**No. of positive dogs**
Lombardo et al. (2012)	Italy	Bone marrow 11.5% (9/78)	SSuRNA (345 bp)	SSuRNA (345-bp)	78
		Lymph node 51.3% (40/78)			
		CS 46.2% (36/78)			
Ferreira et al. (2012)	Brazil	Bone marrow 63.8% (51/80)	kDNA	kDNA	80
		Blood 25% (20/80)			
		CS 83.8% (134/160)			
		Cutaneous tissue 60% (48/80)			
Ferreira et al. (2013)	Brazil	Blood 13.3% (4/30)	kDNA	kDNA	62
		CS 83.3% (25/30)			
		Cutaneous tissue 56.7% (17/30)			
Geisweid et al. (2013)	Germany	Bone marrow 89.5% (17/19)	kDNA	kDNA	43
		CS 81.4% (35/43)			
Pereira et al. (2016)	Brazil	Blood 50% (28/56)	kDNA/120bp	kDNA/120 bp	28
		CS 50% (28/56)			
Selder et al. (2018)	Germany	Blood 75.12% (151/201)	kDNA/120 bp	kDNA	87
		Bone marrow 23.5% (12/51)			
		CS 79.91% (195/244)			
		Lymph node 52.6% (10/19)			
Fernandes et al. (2019)	Brazil	Blood 19.5% (42/215)	kDNA	kDNA	215
		CS 15.3% (33/215)			
Marcelino et al. (2020)	Brazil	Bone marrow 83.1% (54/65)	kDNA	kDNA/145bp	65
		CS 92.3% (60/65)			
		Cutaneous tissue 89.2% (58/65)			
		Lymph node 92.3% (60/65)			
**Total**					**658**

CS: Conjunctival swab; kDNA: kinetoplast minicircle DNA; PCR: polymerase chain reaction (conventional); SSuRNA: small subunit ribosomal RNA; bp: base pairs.

All the studies used PCR as confirmatory test for CVL (n=8), extracting DNA from blood (n=5) and/or lymphoid tissue such as lymph node (n= 3) and bone marrow (n=5). Within those tests, 87.5% of the studies used *kDNA*, 25% used DNA and 12.5% used *SSuRNA* as the target for PCR.

A total population of 658 dogs with confirmed diagnosis of CVL and 951 positive samples composed our dataset, generating the total amount of 2541 PCR results from different samples used in this meta-analysis. Fernandes et al. (2019) was the study with the highest number of dogs evaluated (n=215), while Pereira et al. (2016) was the one with the lowest (n=28).

The articles did not include information about the relationship between the results and qualitative characteristics of the animals, such as age, breed and sex. Moreover, only 50% of the studies included in this meta-analysis described the clinical condition of dogs (symptomatic or asymptomatic).

**Figure 2:**
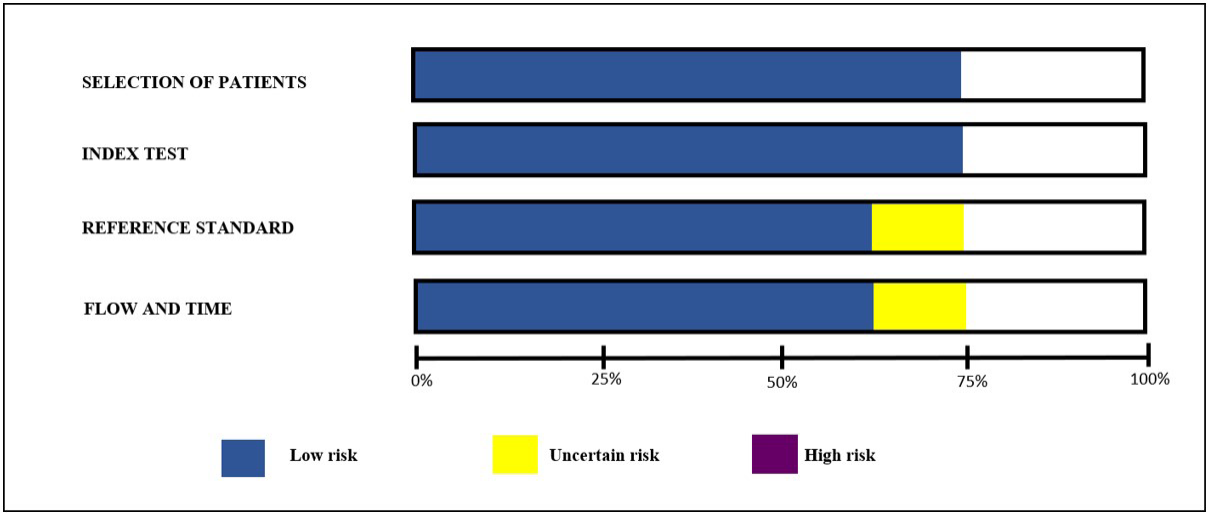
Results of the quality assessment of the studies included in the systematic review using the Quality Assessment of Diagnostic Accuracy Studies 2 (QUADAS-2) tool.

The risk of bias in each study is indicated in Figure 2. It should be noted that one article (Fernandes et al., 2019) provided uncertain results in the risk of bias assessment in the domains of reference standard and of flow and time.

### Meta-analysis

**Figure 3:**
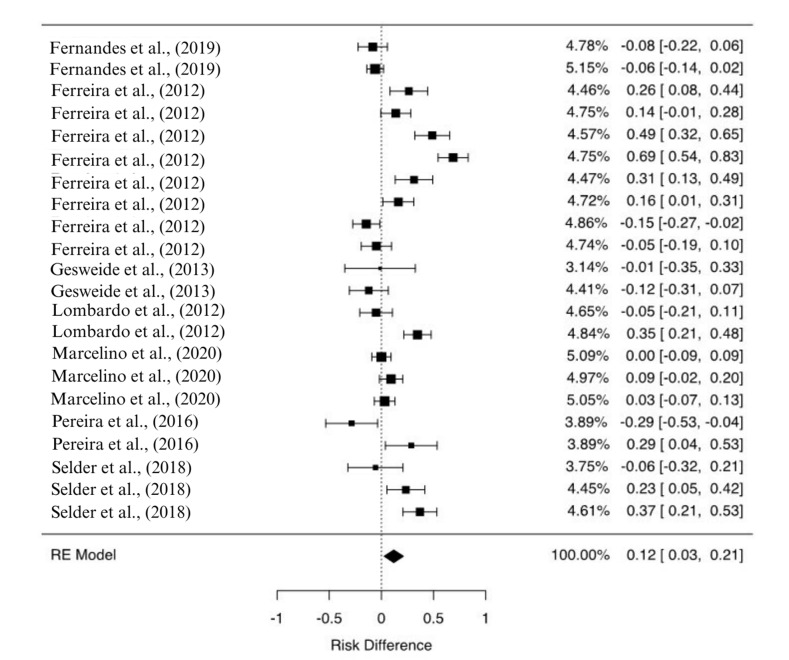
Forest plot of the meta-analysis to determine whether the use of conjunctival swab generates different sensitivity than conventional samples in the diagnosis of canine visceral leishmaniasis by PCR. Main statistics: number of studies (n=8), Fernandes et al., (2019; Ferreira et al., (2012); Ferreira et al. (2013); Geisweid et al. (2013); Lombardo et al., (2012); Marcelino et al., (2020); Pereira et al., (2016); Selder et al., 2018); number of comparisons (k=22); Risk difference and CI 95% (0.119 [0.025-0.212]); Z test (2.49, p=0.013); Tau^2^ (0.0424, p<0.001); I^2^ (88.88%).

The pooled analysis of the risk differences between the intervention and comparison groups showed approximately 12% higher PCR sensitivity (p=0.013) in samples from the intervention group (RD = 0.119 [0.025-0.212]), meaning that, in an overall analysis, there is evidence that the PCR from CS generates higher sensitivity than that from other samples such as blood, skin, lymph nodes and bone marrow. However, significant level of heterogeneity was found between the studies (I^2^: 88,88%, p<0.001), leading to the hypothesis that this overall result might not be replicated at all situations (Figure 3). Therefore, subgroup analyses were performed in order to verify the influence of animals’ clinical condition and sample type/origin on this general result.

When the original dataset was grouped by animals clinical condition (symptomatic, asymptomatic and not described), no significant RDs were observed within the subgroups. When the original dataset was grouped by sample type/origin in the comparison group (blood, lymph node, bone marrow and skin), however, the RD calculated for the comparison of CS and bone marrow (RD = 0.148 [0.032-0.265]) maintained significancy (p=0.012), showing evidence of 14.8% higher PCR sensitivity in samples from the CS group (Table 2). The number of comparisons in each subgroup was very discrepant and, obviously, lower than that obtained in the general analysis.

**Table 2:** Subgroup analyses of the meta-analysis to determine whether the use of conjunctival swab generates different sensitivity than conventional samples in the diagnosis of canine visceral leishmaniasis by PCR.

**Subgroup**	**n**	**k**	**RD [CI, 95%]**	**Z test**	**Tau^2^**	**I^2^**
**Clinical condition**						
sintomatic	4	6	0.180 [-0.064 – 0.425]	1.45 (p=0.148)	0.0863 (p<0.001)	94.32%
assintomatic	4	6	0.123 [-0.110 – 0.356]	1.04 (p=0.300)	0.0731 (p<0.001)	88.56%
not described	4	10	0.0773 [-0.026 – 0.180]	1.47 (p=0.142)	0.022 (p<0.001)	82.82%
**Sample type/origin**						
blood	4	8	0.158 [-0.075 – 0.391]	1.33 (p=0.183)	0.1053 (p<0.001)	95.1%
lymph node	3	3	-0.0167 [-0.092 – 0.059]	-0.431 (p=0.666)	0 (p=0.817)	0%
bone marrow	6	7	0.148 [0.032 – 0.265]	2.50 (p=0.012)	0.0166 (p=0.002)	71.14%
skin	3	4	0.104 [-0.033 – 0.241]	1.49 (p=0.136)	0.0142 (p=0.009)	74.02%

n: number of studies; k: number of comparisons; RD: risk difference; CI: confidence interval; Tau^2^: total heterogeneity; I^2^: inconsistence index.

**Figure 4:**
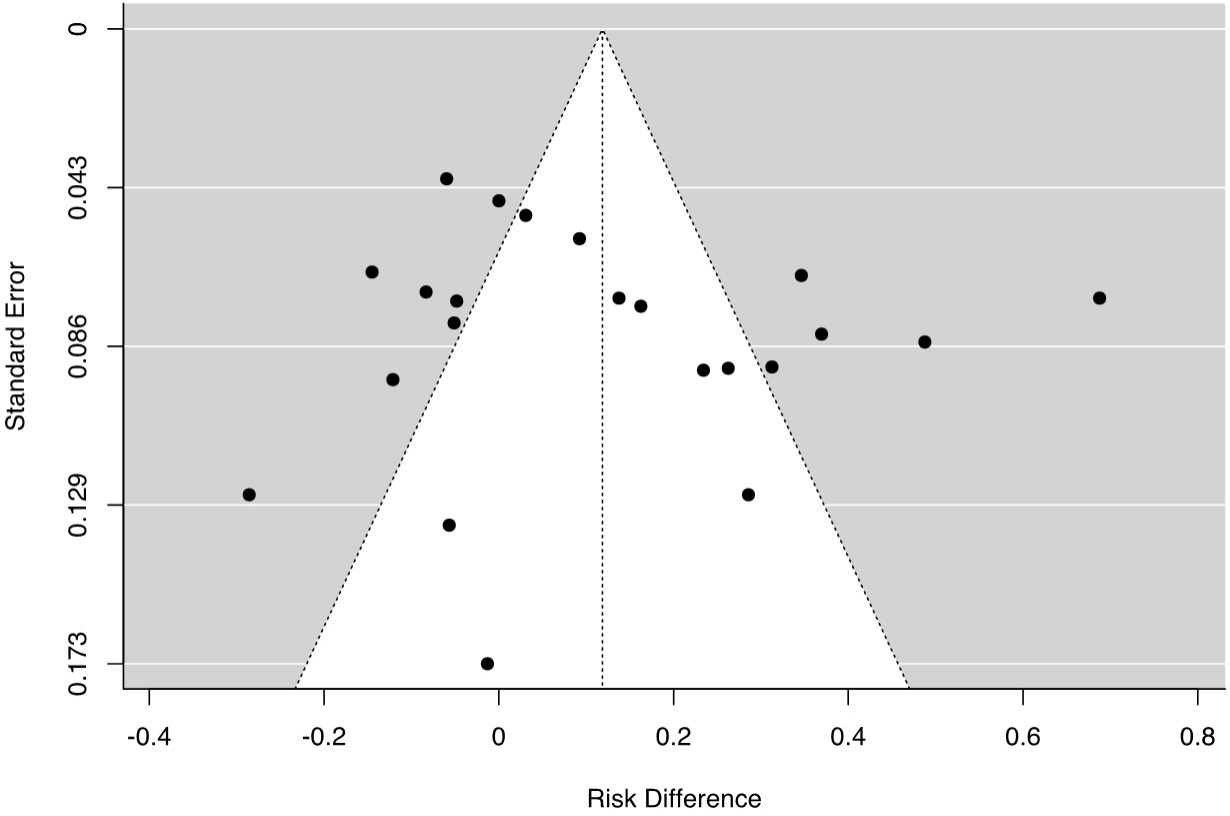
Funnel plot for dataset asymmetry in the meta-analysis to determine whether the use of conjunctival swab generates lower sensitivity than conventional samples in the diagnosis of canine visceral leishmaniasis by PCR. Main statistics: number of studies (n=8); number of comparisons (k=22); Egger regression (p=0.836).

Robustness of the meta-analytical results were tested by the sensitivity analysis. Funnel plot asymmetry was visually investigated and found to be quite irrelevant (Figure 4). To verify this hypothesis, Egger`s regression was performed and confirmed no significant asymmetry between RDs (p=0.836). Thirteen RDs were identified as potential outliers based on their position in the outside area of the normality pyramid of the funnel plot. These studies were excluded, one at a time, from the analysis in order to assess possible changes in the overall result. As no significant changes were detected, these studies were maintained in the analysis.

## Discussion

The comparison of PCR results for the diagnosis of CVL between CS samples and conventional samples reveals a risk difference of approximately 12%, indicating that, in general, CS samples provide 12% better detection of positivity for the disease compared to other types/origins of samples. Therefore, the eye structures should be eligible as a feasible source of samples for the molecular diagnosis of CVL.

Less invasive sampling alternatives for the laboratory diagnosis of CVL, such as CS samples, have been studied extensively in several countries. In this regard, German researchers who tested CS samples by PCR reported 78.4% sensitivity and 93.8% specificity (p<0.05), and considered such samples a good alternative for the diagnosis of CVL (Geisweid et al., 2013). The results of the present meta-analytic study reinforce this statement by generating evidence of higher PCR sensitivity in CS samples than others in the diagnosis of CVL.

Collecting CS samples is not only easy but also costs very little, and offers less discomfort to dogs than conventional sampling, such as skin biopsy, venipuncture and bone marrow puncture (Selder et al., 2018). This low invasiveness also reduces risks inherent to invasive procedures, such as skin biopsy and bone marrow puncture, which require the application of local and general anesthesia, respectively. Moreover, in addition to offering less risk to the health of animals, non-invasive sampling simplifies the disease screening process, reducing the time and costs involved in diagnostic sampling. As a result, screening animals becomes a faster procedure, contributing to the establishment of more assertive measures in CVL control and prevention.

Based on the high heterogeneity identified between studies, subgroup analyses were carried out to verify the influence of animals’ clinical condition and sample type/origin on the general result. Ferreira et al. (2013) pointed out that positive results from CS samples do not depend on the patient’s clinical condition, a statement that was confirmed here, since no different responses were found between the groups of symptomatic and asymptomatic dogs. However, it is important to notice that the number of comparisons (k) within each subgroup was reduced in comparison to that used in the overall analysis. That may imply lower statistical power to detect significant differences between the intervention and comparison groups. Moreover, 50% of the included studies (4 of 8) did not present animals` clinical condition. Therefore, we suggest that future studies subdivide their patients according to their clinical condition so that the relationship between the presence of clinical signs and CVL positivity could be better analyzed.

The type/origin of samples in the comparison group revealed to be influential on the general result. When comparing the PCR sensitivity using CS individually with other samples, CS only remained generating higher sensitivity when compared exclusively with bone marrow. This is somewhat surprising, as bone marrow has traditionally been considered the tissue of choice for the diagnosis of CVL. Although this result is of great relevance to the clinical practice and epidemiological investigation, careful should be used in its interpretation since the number of studies that composed this subgroup was reduced.

The contrasting sensitivity findings between Lombardo et al. (2012) and Geisweid et al. (2013) regarding bone marrow samples in the diagnosis of canine visceral leishmaniasis (CVL) by PCR raise interesting considerations. Lombardo et al. (2012) reported a sensitivity of only 11.5% (9/78), whereas Geisweid et al. (2013) found a sensitivity of 89.5% (17/19) for the same sample type. The different sensitivities reported by Pereira et al. (2016) and Selder et al. (2018) regarding blood samples in the diagnosis of canine visceral leishmaniasis (CVL) using PCR are noteworthy. Pereira et al. (2016) reported a sensitivity of 50% (28/56), whereas Selder et al. (2018) found a sensitivity of only 75.12% (151/201). Furthermore, Fernandes et al. (2019) identified the lowest sensitivity of 19.5% (42 out of 215 samples) for CS samples, whereas Pereira et al. (2016) reported the highest sensitivity of 50% (28 out of 56 samples). Regarding lymph node samples, Marcelino et al. (2020) found a sensitivity of 92.3% (60 out of 65 samples), whereas Lombardo et al. (2012) reported a sensitivity of only 51.3% (40 out of 78 samples). Lastly, Ferreira et al. (2012) reported a sensitivity of 60% (48 out of 80 samples) for cutaneous tissue samples, while Marcelino et al. (2020) found a sensitivity of 89.2% (58 out of 65 samples).

Several factors could contribute to these divergent results. Differences in the study populations, including variations in the prevalence and stage of CVL among the dogs included, might have influenced the sensitivity outcomes. Also, distinct geographical locations with varying CVL prevalence rates, might have led to differences in the parasite burden and consequently in the sensitivity of samples.

In the basis of generating evidence from the published literature, all the results presented here have proved to be robust and, certainly, more precise than the individual estimates from primary studies. However, as science is constantly advancing, new publications might contribute to more accurate estimates of the effect that different samples have on the sensitivity of PCR in the diagnosis of CVL. Therefore, we suggest that researchers continue developing primary studies to compare different samples for the PCR diagnosis of CVL.

It should be noted that the purpose of this study was not to investigate the underlying reasons for the higher sensitivity of CS samples compared to conventional samples. However, future research should be conducted to explore the distinct characteristics of each sample type and elucidate the factors contributing to the enhanced sensitivity of CS samples. A comprehensive narrative review of existing literature summarizing the characteristics of each sample type could serve as a solid foundation for such investigations.

Ultimately, the paucity of data in some published studies, such as the sex of the dogs under study and detailed descriptions of their clinical signs, limited the scope of the subgroup analysis. Thus, we recommend that researchers in this field include descriptions of the characteristics of patients in their studies, thus enabling analyses that correlate these characteristics with others of interest. Although the results of this meta-analysis are promising, it is important to emphasize that further research is needed to validate and improve the use of CS samples in the diagnosis of CVL by PCR. Future studies should explore other aspects, such as the variability of sensitivity at different stages of infection, comparison with other diagnostic techniques, and analysis of specific patient subgroups.

## Conclusions

There is evidence in the literature that the use of CS samples generates 12% higher sensitivity than conventional samples (blood, skin, lymph node and bone marrow) in the diagnosis of CVL by PCR. Thus, CS sampling offers a promising alternative to the molecular diagnosis of CVL, since it is easy and fast, minimally invasive and inexpensive compared to conventional samples, in addition to favoring the PCR detection of the disease. However, additional studies may be required to evaluate the diagnostic efficacy of CS compared to each conventional sample individually.
